# Cotton Rats and House Sparrows as Hosts for North and South American Strains of Eastern Equine Encephalitis Virus

**DOI:** 10.3201/eid1609.100459

**Published:** 2010-09

**Authors:** Nicole C. Arrigo, A. Paige Adams, Douglas M. Watts, Patrick C. Newman, Scott C. Weaver

**Affiliations:** Author affiliations: University of Texas Medical Branch, Galveston, Texas, USA (N.C. Arrigo, A.P. Adams, P.C. Newman, S.C. Weaver);; University of Texas at El Paso, El Paso, Texas, USA (D.M. Watts)

**Keywords:** Eastern equine encephalitis, alphavirus, arbovirus, Sigmodon, cotton rats, house sparrows, Passeriformes, viruses, research

## Abstract

*Eastern equine encephalitis virus* (EEEV; family *Togaviridae*, genus *Alphavirus*) is an arbovirus that causes severe disease in humans in North America and in equids throughout the Americas. The enzootic transmission cycle of EEEV in North America involves passerine birds and the ornithophilic mosquito vector, *Culiseta melanura*, in freshwater swamp habitats. However, the ecology of EEEV in South America is not well understood. *Culex (Melanoconion)* spp. mosquitoes are considered the principal vectors in Central and South America; however, a primary vertebrate host for EEEV in South America has not yet been identified. Therefore, to further assess the reservoir host potential of wild rodents and wild birds, we compared the infection dynamics of North American and South American EEEV in cotton rats (*Sigmodon hispidus*) and house sparrows (*Passer domesticus*). Our findings suggested that each species has the potential to serve as amplification hosts for North and South America EEEVs.

## Abstract 2

TOC summary: Wild rodents and wild birds can serve as amplification hosts.

*Eastern equine encephalitis virus* (EEEV; family *Togaviridae*, genus *Alphavirus*) is an arbovirus that causes severe neurologic disease in humans in North America and in equids throughout the Americas (*1*). EEEV strains that circulate in North America and the Caribbean (NA EEEV, lineage I) are distinguishable from those that circulate in Central and South America (SA EEEV, lineages II–IV) by the following: antigenicity (4 distinct subtypes), genetics (20%–25% nt sequence divergence), phylogenetic and evolutionary patterns, epidemiology, human pathogenicity, and geographic distribution (*2*). One theory for their markedly different characteristics is that EEEV adapted to a unique North American ecologic niche after its introduction and evolutionary divergence from EEEV in Central and South America (*3*). Although the ecology of vectors and vertebrate hosts for NA EEEV has been well defined, the ecology for SA EEEV remains poorly characterized, which limits our understanding of the divergence of these viruses.

Enzootic circulation of EEEV in eastern North America is primarily supported by a variety of avian reservoirs in the order Passeriformes and by the highly ornithophilic mosquito vector, *Culiseta melanura*, in freshwater swamp habitats. However, under favorable amplification conditions, sporadic epizootic and epidemic transmission occurs by bridge vectors (e.g., *Aedes* spp. mosquitoes) that have more catholic feeding behaviors. These vectors have the ability to broaden the virus’ amplification host range to other avian or mammalian species in habitats that pose greater risk for incidental hosts, such as humans and equids. For example, recent studies in some southeastern foci of North America suggest that enzootic and/or epizootic EEEV transmission may involve ectothermic hosts (e.g., reptiles and amphibians) and herpetophilic mosquito vectors (*4*). Rodents have not been implicated in transmission of enzootic NA EEEV; however, seroprevalence data (*5*) support their susceptibility to infection and warrant consideration of their potential to serve as vertebrate hosts during epizootic transmission.

Isolation of SA EEEV from *Culex (Melanoconion)* spp. mosquitoes in the Spissipes section (e.g., *Cx. pedroi*, *Cx. taeniopus*) suggests that they are the principal enzootic, and potentially epizootic, mosquito vectors (*6**–**8*) in Central and South America. These mosquito species have broad host preferences—mammalian, avian, and reptilian (*9*)—but the primary vertebrate host for SA EEEV has not yet been identified. Virus isolations and seroprevalence data demonstrate that wild birds, rodents, marsupials, and reptiles are susceptible to infection (*6**,**10**–**12*). However, the involvement of these vertebrates in the enzootic transmission of SA EEEV remains unclear.

Venezuelan equine encephalitis virus (VEEV) is the closest genetic relative to EEEV and circulates sympatrically with SA EEEV. Like SA EEEV, *Culex (Melanoconion)* spp. mosquitoes serve as the primary enzootic vectors of VEEV (*13**–**15*). Small mammals are the principal reservoir hosts of VEEV (*15*), although a wide variety of vertebrate species have antibodies against VEEV (*16**,**17*). Phylogenetic comparisons of SA EEEV and enzootic VEEV subtypes ID and IE have shown similar patterns of evolution that are consistent with the use of mammalian vertebrate hosts rather than the avian hosts involved in NA EEEV transmission (*2*). Therefore, the similarities in geographic range, vector ecology, and phylogenetic profiles of SA EEEV and VEEV support the hypothesis of similar mammalian vertebrate host usage, unlike the avian host usage for NA EEEV.

To further test this hypothesis of differential vertebrate hosts for NA versus SA EEEV strains, we compared their infection dynamics in a wild rodent (cotton rat, *Sigmodon hispidus*) known to support VEEV transmission and in a passerine bird (house sparrow, *Passer domesticus*) known to be a competent host of NA EEEV. Our goals were to better understand the ecology of SA EEEV, which will help clarify the extent to which these viruses have ecologically diverged and the parameters contributing to or limiting the potential emergence or adaptation of EEEV in naive environments.

## Materials and Methods

### Animals

During August and September 2007, cotton rats (*S. hispidus berlandieri*) (*18*) were collected from Galveston Island State Park, Texas, USA (29.27°N, 94.83°W) by using live-capture traps (H.B. Sherman Traps, Tallahassee, FL, USA). The weights of the feral rats ranged from 52 to 138 g, suggesting a wide range of ages (*19*). Laboratory-born progeny of captured rats were also used in experiments for a total of 3 cohorts: feral, 7–8-wk progeny, and juvenile (2–3 wk progeny). House sparrows were collected by using mist nets throughout Houston, Texas. Birds were morphologically identified, sexed, and aged (hatch-year vs. after hatch-year). To determine viremia and antibody responses, we experimentally infected 2 cohorts, collected in June and July 2008. To determine survival rates without manipulation, we infected a third cohort, collected in July and August 2009. All experimental groups of rats and sparrows were matched for sex and approximate age or life stage.

Animals were transported directly to the BioSafety Level 3 facility at the University of Texas Medical Branch, housed individually, and given food and water ad libitum. During acclimation, feral rats were determined to be seronegative for EEEV, VEEV, and western equine encephalitis virus by 80% plaque-reduction neutralization tests (PRNT_80_), and they were screened by immunofluorescent assay for persistent infection with Bayou (*Hantavirus*) and Whitewater Arroyo viruses (*Arenavirus)*, known to be enzootic in the region. Hemagglutination inhibition tests also determined that the sparrows were seronegative for EEEV and western equine encephalitis virus, as well as for the flaviviruses St. Louis encephalitis virus and West Nile virus. All studies were approved by the Institutional Animal Care and Use Committee at the University of Texas Medical Branch.

### Virus Isolation and Animal Infection

NA EEEV strain FL93-939 (NA FL93, lineage I) was isolated from a *Culex* spp*.* mosquito pool in Florida in 1993, cloned into cDNA form (*20*), and rescued from baby hamster kidney cells. SA EEEV strains 77U1104 (SA PE70, lineage II) and C49 (SA CO92, lineage III) were isolated from sentinel hamsters in Peru, 1970, or Columbia, 1992, respectively, and passaged once in Vero cells.

We inoculated each animal subcutaneously in the thigh with virus or with uninfected medium for negative controls (Table 1). The target dose was ≈3 1og_10_ PFU, which is consistent with the approximate maximum amount introduced by the bite of an alphavirus-infected mosquito (*21*). Animals were monitored daily for signs of illness and killed when moribund or ≈4 wk postinfection. For viremia and antibody assays, 100-µL blood samples were collected from the retroorbital sinus of rats or from the jugular vein of sparrows for the first 5–7 d postinfection. To determine seroconversion status, we also collected samples on days 29–30 for rats and days 14, 22, 24, and/or 39 for sparrows. To reduce handling, we randomly divided the sparrow cohorts into 2 groups from which blood was collected on alternate days. Blood from rats was collected daily.

**Table 1:** Total cohort sizes and eastern equine encephalitis virus inoculum*

Characteristic	Eastern equine encephalitis virus strain								No. controls
	FL93-939 (FL93)			77U1104 (PE70)			C49 (CO92)		
No.	Dose, log_10_ PFU	No.	Dose, log_10_ PFU	No.	Dose, log_10_ PFU
Rat cohort									
Juvenile	6	3.1		6	3.5		NT	NT	1
Mature	8	2.2–3.1		13	3.8–4.2		12	2.8–3.3	4
House sparrow cohort*									
Infection	13	2.9–3.6		13	2.8–3.8		13	3.9–4.9	4
Nonmanipulation	23	2.9		23	3.2		22	3.4	13

*Total no. animals in nonmanipulation cohort also includes animals from infection cohort. NT, not tested.

### Virus Titer and Antibody Assays

Blood samples were immediately diluted 1:10 with phosphate-buffered saline supplemented with 10% heat-inactivated fetal bovine serum and penicillin (10,000 U/mL), streptomycin (10,000 µg/mL), and gentamicin (50 mg/mL). Diluted whole blood was tested to determine virus titers by plaque assay and antibody titers (maximum dilution 1:1,280) by PRNT_80_ on Vero cells, as described (*22*). Diluted serum samples from >14 d postinfection were also tested for antibodies by PRNT_80_ (*22*).

### Data and Statistical Analyses

Only those animals with evidence of infection (detection of virus or antibodies) were included in the statistical analyses. Viremia and antibody response profiles were determined by calculating daily geometric mean titer values. Viremia and antibody values below the limit of detection were considered halfway between 0 and limit of detection: 1.0 log_10_ PFU/mL for viremia and 1:20 neutralizing antibody. A 2-way analysis of variance with Bonferonni posttest was used to analyze viremia and antibody data. Although all cohorts were considered individually for these analyses, the feral and 7-8–week rat cohorts and the 2 sparrow cohorts were each combined for graphical clarity and because their daily mean viremia titers and survival rates did not differ statistically. House sparrow survival analysis also included a third cohort for which we assessed survival rates in those not manipulated. These combined groups are denoted mature cotton rats and house sparrows. We used the log-rank test to analyze survival data. p<0.05 was considered significant.

## Results

### Viremia Profiles

#### Within Species

**Figure 1:**
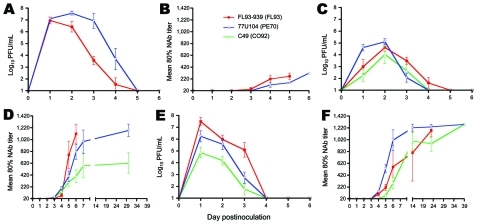
Mean viremia (A–C) and neutralizing antibody response (D–F) profiles in juvenile cotton rats (A, D), mature cotton rats (B, E), and house sparrows (C, F) after subcutaneous inoculation with 3–4 log_10_ PFU of North American eastern equine encephalitis virus (EEEV) strain FL93 (red lines), South American (SA) EEEV strain PE70 (blue lines), or SA EEEV strain CO92 (green lines). Note the difference in scale of the x-axis for the antibody response of juvenile rats. NAb, neutralizing antibody. Error bars represent SEM.

The viremia profiles of mature cotton rats showed higher initial replication of SA PE70 than NA FL93 and SA CO92, a trend particularly evident 24 h postinfection (Figure 1, panel C). All titers peaked by 48 h; SA PE70 generated the highest titers among mature rats and sharply declined thereafter. Although not statistically significant (Table 2), peak titers of NA FL93 and SA CO92 were lower than titers of SA PE70 and declined less rapidly through 72 h postinfection. The trend for juvenile rats was also higher titers of SA PE70 than of NA FL93 (Figure 1, panel A); titers for each virus strain remained significantly higher for juvenile than for mature rats (p<0.001, Tables 2, 3). NA FL93 peaked by 24 h postinfection; titers of SA PE70 were similar at 24 h, surpassed NA FL93 by 48 h, and continued to be significantly higher (p<0.001) through 96 h postinfection. SA PE70 viremia in the juvenile rats was the highest among all virus strains and rat cohorts.

**Table 2:** Comparisons of mean peak titers of eastern equine encephalitis virus within experimental cohorts*

Cohort	Mean peak viremia titer, log_10_ PFU/mL (± SEM)				Within-cohort comparison, p value		
	FL93 (FL93-939)	PE70 (77U104)	CO92 (C49)		FL93 vs. PE70	FL93 vs. CO92	PE70 vs. CO92
Juvenile cotton rats	7.0 (0.3)	7.6 (0.2)	Not tested		0.089	NT	NT
Mature cotton rats	4.5 (0.3)	5.1 (0.3)	3.8 (0.8)		0.140	0.374	0.078
House sparrows	7.5 (0.4)	6.2 (0.5)	4.9 (0.4)		0.051	**<0.001**	0.060

*Two-tailed p-values determined by Student *t* test; p values <0.001 are not specified. **Boldface** indicates significant difference. NT, not tested.

**Table 3:** Comparisons of mean peak titers of eastern equine encephalitis virus between experimental cohorts*

Virus	Mean peak virus titer, log_10_ PFU/mL (± SEM)				Between-cohort comparison, p value		
	Juvenile cotton rats	Mature cotton rats	House sparrows		Juvenile vs. mature rats	Juvenile rats vs. house sparrows	Mature rats vs. house sparrows
FL93 (FL93-939)	7.0 (0.3)	4.5 (0.3)	7.5 (0.4)		**<0.001**	0.271	**<0.001**
PE70 (77U104)	7.6 (0.2)	5.1 (0.3)	6.2 (0.5)		**<0.001**	**0.026**	**0.036**
CO92 (C49)	NT	3.8 (0.8)	4.9 (0.4)		NT	NT	NT

*Two-tailed p values determined by Student *t* test; p values <0.001 are not specified. **Boldface** indicates significant difference. NT, not tested.

House sparrows supported higher NA FL93 replication than SA PE70 throughout the experiment; SA CO92 replication was the lowest (Figure 1, panel E). NA FL93 and SA CO92 viremia profiles were similar between the 2 sparrow cohorts; however, SA PE70 titers were slightly higher in the second sparrow cohort (data not shown, differences not significant). The titers of all virus groups peaked by 24 h; the highest peak titers were in the NA FL93 infection groups (Table 2). NA FL93 and SA PE70 titers were similar at 48 h; however, NA FL93 titers were 1–3 logs higher than SA PE70 and SA CO92 at 24 and 72 h postinfection.

#### Between Species

Rats and sparrows were susceptible to infection with all EEEV strains; however, trends in NA and SA EEEV viremia profiles were opposite between species (Figure 1). In rats, SA PE70 titers were highest, but in sparrows, NA FL93 titers were highest. SA CO92 replication was lowest overall, and peak viremia titers were comparable between species. Viremia in mature rats peaked at 48 h postinfection and in sparrows peaked at 24 h postinfection. This rapid initial replication in sparrows also corresponded to significantly higher peak titers of NA FL93 (p<0.05–0.001) compared with those of mature rats (Table 3). SA PE70 titers were also generally higher in sparrows than in mature rats. SA CO92 titers were marginally higher in the sparrows than in mature rats; however, differences in their peak titers were not significant. In contrast, the viremia titers in juvenile rats were similar to or higher than those in sparrows. Juvenile rats sustained significantly higher SA PE70 viremia titers than the sparrows at 48, 72, and 96 h postinfection (p<0.01–001), but NA FL93 titers were comparable on all days.

### Survival Rates

**Figure 2:**
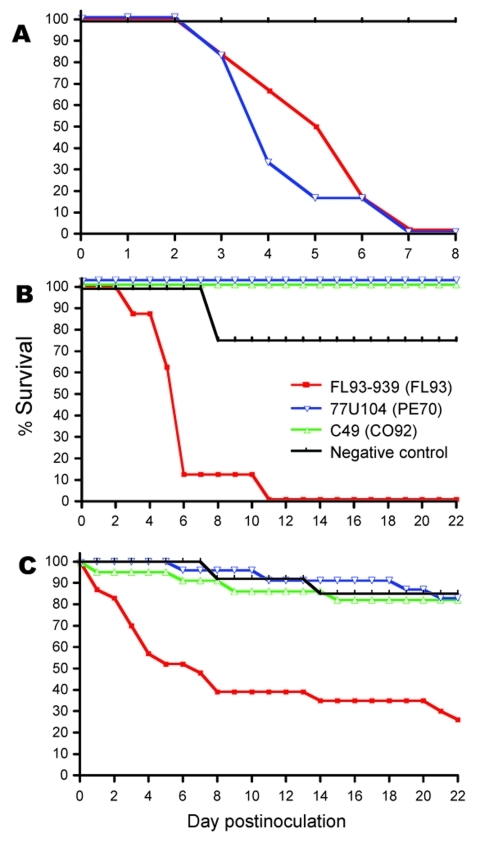
Survival rates for juvenile cotton rats (A), mature cotton rats (B), and house sparrows (C) after subcutaneous inoculation with ≈3–4 log_10_ PFU of North American eastern equine encephalitis virus (EEEV) strain FL93 (red lines), South American (SA) EEEV strain PE70 (blue lines), or SA EEEV strain CO92 (green lines). Survival rates beyond day 22 postinfection did not differ. Experimental infection of juvenile cotton rats with SA EEEV strain CO92 was not conducted.

Of 25 mature cotton rats infected with either SA PE70 or SA CO92, 100% survived and had no signs of disease (Figure 2, panel B). In contrast, all mature rats infected with NA FL93 died. Signs of illness began on day 4 postinfection; by day 6, most animals exhibited lethargy, anorexia, dehydration, and neurologic manifestations of instability and erratic movement. Most mature rats died during days 3–6, and 1 rat died on day 17 after a prolonged illness with anorexia. One uninfected control animal died on day 7 without any detectable signs of illness. None of the juvenile rats infected with either SA PE70 or NA FL93 survived; their illness was similar to that observed in mature rats infected with NA FL93 (Figure 2, panel A). All juveniles died during days 3–6, and the mean time to death did not differ significantly between groups. Juvenile rats were not inoculated with SA CO92.

For sparrows, NA FL93-infection resulted in a 26% survival rate, which was significantly lower than the 82%–83% survival rates for SA PE70- and SA CO92–infected sparrows (p<0.001). Mortality rates for sparrows did not differ significantly from those for mature rats for all viruses (p>0.3). The NA FL93–induced mortality rate for juvenile rats was comparable to those for NAE FL93–infected sparrows and mature rats (p>0.3); however, the mortality rate for juvenile rats infected with SA PE70 was significantly greater than that for sparrows and mature rats infected with SA PE70 (p<0.001).

### Antibody Responses

For rats and sparrows, antibodies were detected by day 4 postinfection (Figure 1, panels B, D, and F). Antibodies were detected in all animals that had detectable viremia and that survived beyond day 3; however, some mature rats infected with SA CO92 had low antibody titers in the absence of detectable viremia. In the mature rats, the antibody response to NA FL93 was initially more robust than that to SA EEEV, but SA EEEV antibodies were detected 1–2 days earlier (Figure 1, panel D). Similar to the pattern in mature rats, juvenile rat titers in response to NA FL93 were initially higher than titers in response to SA PE70, although juvenile rat antibody responses were much lower overall (Figure 1, panel B). The antibody responses of sparrows showed the opposite pattern to those of rats (Figure 1, panel F). Although titers were similar to those of mature rats, SA PE70–infected sparrows generated a more robust initial response than those infected with NA FL93 or SA CO92. Unlike the mature rates, some NA FL93–infected sparrows survived, and the antibody response to all 3 viruses ultimately reached the highest measured titers. The early antibody responses to NA FL93 and SA PE70 in mature rats and in sparrows were inversely related to their respective viremia profiles; however, a consistent correlation at the individual animal level was not found.

## Discussion

Reservoir host competence depends primarily on an animal’s susceptibility to infection, the intensity of viremia, and the duration of viremia sufficient to infect appropriate mosquito vectors. Rats and sparrows were equally susceptible to infection with the NA and SA EEEV strains and doses used in this study, and their viremia lasted 4–5 days. However, the patterns of infection differed; the general trend was higher SA PE70 replication in rats and higher NA FL93 replication in sparrows, consistent with the hypothesis that SA EEEV strains use mammalian hosts as their principal reservoirs. Infections of both adult species with SA CO92 resulted in the lowest overall viremia and antibody titers, suggesting an overall attenuation of this strain.

The minimum infectious oral dose for *Cs. melanura* mosquitoes, the primary NA enzootic vector, corresponds to a viremia of ≈3 log_10_ PFU/mL, and almost all mosquito species infected experimentally become infected after blood meals of at least 6 log_10_ PFU/mL (*23**–**25*). Regardless of slight variations in inoculum doses, all viruses resulted in viremia titers in rats and sparrows high enough to infect enzootic and epizootic vectors in North America. The highest and longest titers of NA EEEV were found in sparrows and of SA PE70 were found in juvenile cotton rats. Although the preferred habitats of both animal species differ from the hardwood swamps inhabited by *Cs. melanura* mosquitoes, our results suggest that both species have the potential to play a role as amplification hosts during epizootic and epidemic transmission. Although mosquito vectors in North America have not been evaluated for their competence to transmit the SA EEEV strains we tested, the productive infection of both animal species we tested highlights the potential for SA EEEV emergence in North American habitats.

Only 1 study has assessed the vector competence of mosquitoes for EEEV in South America (*8*). Turell et al. (*8*) observed that at least 50% of mosquito species in Peru, including the presumed local enzootic mosquito vector, *Cx. pedroi*, became infected after feeding on chickens or hamsters that had moderate levels of viremia (4.6–5.8 log_10_ PFU/mL), and even more species became infected after ingesting higher doses from blood meals (7.7–8.5 log_10_ PFU/mL). Given these limited data, our study indicates that viremia sufficient in intensity and duration to serve as a source of infection for mosquito vectors in South America develops in sparrows and cotton rats. Additional vector-competence experiments with species from other foci of enzootic SA EEEV transmission (e.g., *Cx. taeniopus* mosquitoes) and experimental infections of sympatric animal species would help confirm these results and provide a more complete understanding of the ecology of EEEV in South America.

Although survival is not an essential requirement for host competence, the infection profile and pathogenicity of a virus in a host can be indicative of the host’s evolutionary history. The higher virus titers induced by SA PE70 and the survival of all mature cotton rats after infection by both SA EEEV strains may indicate selection for resistance to disease or selection for attenuation of these SA viruses in this species. Selection for resistance to disease has been proposed to explain the benign outcome of experimental infections of various rodents with sympatric VEEV (*18**,**26**,**27*) as opposed to the severe disease outcome for closely related rodents from regions where the virus is not endemic. Although the subspecies of cotton rats (*S. hispidus berlandieri*) collected in Galveston does not reside sympatrically with SA EEEV, it is genetically and geographically close to members of the *S. hispidus* rat complex in areas of enzootic SA EEEV transmission (e.g., *S. hispidus hirsutus* rats) (*28*). The results of our study could reflect a long-term association between SA EEEV and ancestral *S. hispidus* rats and support their potential role in enzootic transmission of EEEV in South America.

Unlike mature rats, juvenile cotton rats experienced severe neurologic disease and 100% mortality rates after infection with either NA FL93 or SA PE70. These age-dependent disease and mortality rates have been previously observed with Sindbis virus (another alphavirus) and EEEV infection of laboratory mice (*29**,**30*). Explanations include increased virus replication in immature neurons (*31*) and metabolically active osteoblasts (*32*) and potential involvement of differential interferon induction and response (*33*). Gardner et al. (*29*) observed age-dependent survival of mice after subcutaneous inoculation with an adult mouse–attenuated strain of SA EEEV (BeAr 436087); however, NA FL93-939 resulted in severe disease and death for mice of all ages (*29*). These observations are consistent with the results of our experimental infections of mature and juvenile rats.

The survival profiles between sparrows and mature rats after experimental infection with NA or SA EEEV were similar. Although both SA strains caused slightly higher mortality rates for sparrows than for mature rats, these differences were not significant. Sparrow deaths resulting from NA FL93 correlated with the development of extremely high peak viremia titers at 1 day postinfection, suggesting the inability to control early virus replication. Although the SA EEEV virus titers at 1 day postinfection were higher in sparrows than in mature cotton rats, peak titers remained comparable between species, and no significant differences in survival rates were noted. In addition, all rats infected with NA FL93 died, despite relatively low peak viremia in mature rats. These observations suggest underlying differences in the pathogenesis of NA and SA EEEV within each species that go beyond their relative susceptibility to virus infection.

The NA EEEV-induced deaths of sparrows may also reflect the relatively recent introduction of these birds into the United States and their shorter history of exposure to EEEV. Komar et al. (*23*) reported similar mortality rates and correlation with peak viremia in NA EEEV experimental infections of European starlings (*Sturnus vulgaris*), also a nonnative species introduced into the United States in the late 1800s (*23*). Many domesticated captive birds, such as whooping cranes (*34*), emus (*35*), and ring-neck pheasants (*36*), as well as native free-ranging wild birds such as American crows (*Corvus brachyrhynchos*) (*37*) and blue jays (*Cyanocitta cristata*) (*38*), have also reportedly experienced severe disease and high mortality rates in response to EEEV infection. However, seroprevalence of EEEV antibodies in surviving wild birds in North (*39**,**40*) and South America (*11**,**12*) indicates that some avian species have the ability to survive natural infection.

Although additional ecologic studies are needed to confirm a primary vertebrate host for EEEV in Central and South America, our results demonstrate the competence of rats and of sparrows to serve as amplification hosts for NA and SA EEEV. However, the lack of detectable disease in mature rats after SA EEEV infection supports the possibility of long-term exposure of rodents to EEEV in South America. This dichotomy in rat survival rates should also be explored as a potential model for studying differences in NA and SA EEEV viral tropism and pathogenesis, which may explain differences in virulence for humans. Although enzootic transmission of NA EEEV primarily involves passerine birds, the relative competence of cotton rats and sparrows as NA EEEV hosts highlights the probable influence of *Cs. melanura* mosquito habitats and avian host preferences in shaping the ecology of EEEV in North America. NA and SA EEEV experimental infections of vertebrate and mosquito species from regions of enzootic SA EEEV transmission would complement these studies and broaden our understanding of the evolution of these viruses and their potential to emerge and adapt to new environments.
